# P-1035. Hypo- and hyper-inflammatory phenotypes both predict adverse clinical outcomes in patients with presumed and confirmed *Pneumocystis jirovecii* pneumonia without underlying human immunodeficiency virus infection

**DOI:** 10.1093/ofid/ofae631.1225

**Published:** 2025-01-29

**Authors:** Matthew C Y Koh, Jinghao Nicholas Ngiam, Paul Tambyah, Lionel H W Lum

**Affiliations:** National University Health System, Singapore, Singapore; National University Health System, Singapore, Singapore; National University Hospital, Singapore, Singapore, Not Applicable, Singapore; NUHS, Singapore, Not Applicable, Singapore

## Abstract

**Background:**

With the advent of highly effective antiretroviral therapies, the burden of *Pneumocystis jirovecii* pneumonia (PJP) has shifted from people living with human immunodeficiency virus (HIV) to other immunocompromised hosts. We hypothesize that amongst patients with PJP without underlying HIV, there are unique hypo- and hyper-inflammatory phenotypes that may predict clinical outcomes.

Clinical characteristics of patients with PJP without underlying HIV, stratified by inflammatory phenotypes

**Figure:**
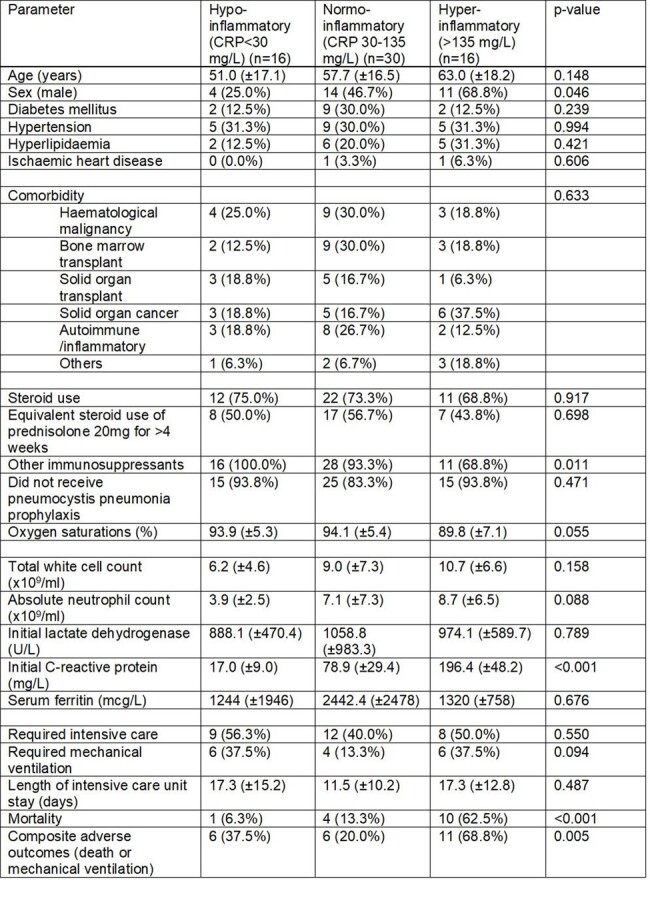

Clinical characteristics of patients with PJP without underlying HIV, stratified by inflammatory phenotypes

**Methods:**

Consecutive patients with a diagnosis of PJP and a negative HIV test admitted to a tertiary hospital in Singapore were examined from 2006-2023. We included patients who had a C-reactive protein (CRP) measured within 48 hours of admission. The cohort was then divided into three groups stratified by CRP centiles, namely the hypo-inflammatory (up to 25^th^ centile), normo-inflammatory (25^th^ to 75^th^ centile) and hyper-inflammatory (above 75^th^ centile) groups. Clinical profile and outcomes were compared amongst the three groups. A composite adverse outcome consisting of in-hospital mortality or need for mechanical ventilation was evaluated.

Multivariable analysis showing predictors of adverse outcome (death or mechanical ventilation) in patients with PJP without underlying HIV

**Figure:**
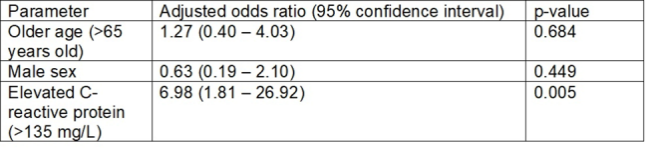

**Results:**

A total of 62 patients were examined, of which 16 were in the hypo-inflammatory group (CRP< 30 mg/L), 32 were in the normo-inflammatory group (CRP 30-135 mg/L) and the remaining 16 were in the hyper-inflammatory group (CRP > 135 mg/L). Adjunctive corticosteroid use was also similar across the three groups. Importantly, mortality was highest in the hyper-inflammatory group (6.3 vs 13.3 vs 62.5%, p< 0.001). However, in terms of composite adverse outcome, both the hypo-inflammatory (37.5%) and hyperinflammatory groups (68.8%) had higher prevalence of adverse outcomes than the normo-inflammatory group (20.0%, p=0.005). On multivariable analyses, after adjusting for age and sex, the hyperinflammatory phenotype remained independently associated with adverse outcomes (adjusted OR 6.98, 95%CI 1.81 – 26.92, p=0.005)

**Figure:**
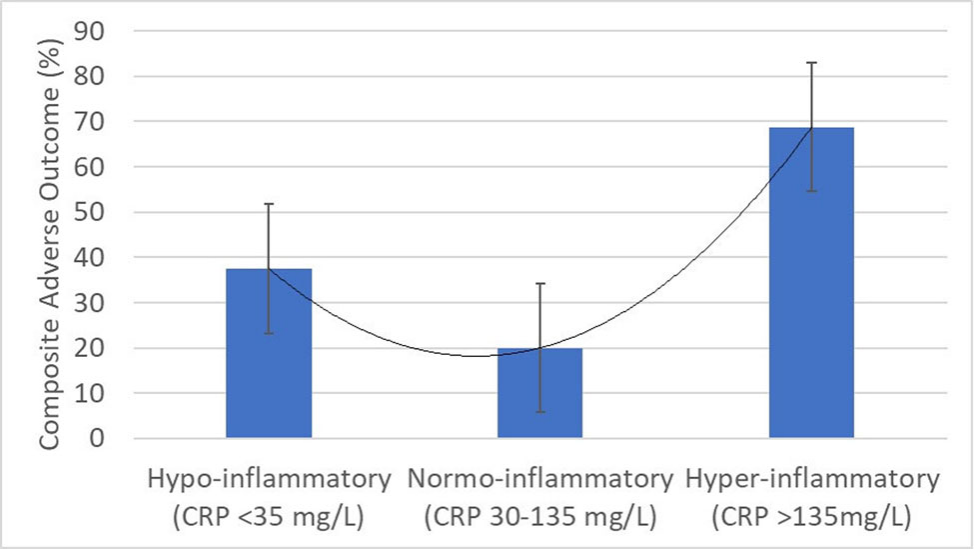

Composite adverse outcomes (death or mechanical ventilations) across the three groups of hypo-, normo- and hyper-inflammatory patients with PJP without underlying HIV

**Conclusion:**

There may be a U-shaped relationship between C-reactive protein and clinical outcomes in patients with PJP without underlying HIV infection. Patients from both ends of the inflammatory spectrum had increased adverse outcomes in our study. These findings invite further study on the influence of degree of immunosuppression on subsequent inflammation and adverse outcomes among non-HIV infected immunocompromised hosts with PJP.

**Disclosures:**

**Paul Tambyah, MBBS (S'pore), Diplomate, American Board of Internal Medicine and Infectious Diseases**, Moderna: Grant/Research Support|Sanofi-Pasteur: Grant/Research Support

